# Model to Predict Overall Survival in Patients With Hepatocellular Carcinoma After Curative Hepatectomy

**DOI:** 10.3389/fonc.2020.537526

**Published:** 2021-03-05

**Authors:** Li-xiang Zhang, Pan-quan Luo, Lei Chen, Dong-da Song, A-man Xu, Peng Xu, Jia Xu

**Affiliations:** ^1^ Department of General Surgery, The First Affiliated Hospital of Anhui Medical University, Hefei, China; ^2^ Department of General Surgery, The Fourth Affiliated Hospital of Anhui Medical University, Hefei, China; ^3^ Department of General Surgery, First Affiliated Hospital of Wenzhou Medical University, Wenzhou, China; ^4^ Department of Hepatobiliary Surgery, Xiang’an Hospital of Xiamen University, Xiamen, China; ^5^ Oncology Department, Beijing University of Chinese Medicine Third Affiliated Hospital, Beijing, China

**Keywords:** hepatocellular carcinoma, prognosis, nomogram, overall survival, model

## Abstract

**Background:**

The prognosis of patients with hepatocellular carcinoma (HCC) remains difficult to accurately predict. The purpose of this study was to establish a prognostic model for HCC based on a novel scoring system.

**Methods:**

Five hundred and sixty patients who underwent a curative hepatectomy for treatment of HCC at our hospital between January 2007 and January 2014 were included in this study. Univariate and multivariate analyses were used to screen for prognostic risk factors. The nomogram construction was based on Cox proportional hazard regression models, and the development of the new scoring model was analyzed using receiver operating characteristic (ROC) curve analysis and then compared with other clinical indexes. The novel scoring system was then validated with an external dataset from a different medical institution.

**Results:**

Multivariate analysis showed that tumor size, portal vein tumor thrombus (PVTT), invasion of adjacent tissues, microvascular invasion, and levels of fibrinogen and total bilirubin were independent prognostic factors. The new scoring model had higher area under the curve (AUC) values compared to other systems, and the C-index of the nomogram was highly consistent for evaluating the survival of HCC patients in the validation and training datasets, as well as the external validation dataset.

**Conclusions:**

Based on serum markers and other clinical indicators, a precise model to predict the prognosis of patients with HCC was developed. This novel scoring system can be an effective tool for both surgeons and patients.

## Introduction

Primary hepatocellular carcinoma (HCC) is one of the most prevalent malignant tumors and ranks as the second leading cause of cancer-related death worldwide (1). Surgery is the most effective treatment for the majority of patients and provides an opportunity for longer survival times. However, the cancer recurrence rate and prognosis are still not optimistic even after radical resection, and the 5-year survival rates remain poor (2). Due to its high mortality, HCC remains an important public health burden in China (3). Therefore, it is urgent to further explore the precise markers that can evaluate the progression and prognosis of HCC.

There are many scoring systems with significant value for predicting the prognosis of HCC patients. The internationally accepted systems for evaluating the prognosis and progression of HCC include the Barcelona Clinic Liver Cancer (BCLC) staging system, the Okuda staging system (Okuda), the Cancer of the Liver Italian Program (CLIP) and the seventh edition of the TNM (TNM seventh) system (4–6). In recent years, serum markers that reflect liver function have received more attention. Among them, a simple model based on gamma-glutamyl transpeptidase (GGT) and platelets can predict survival in hepatitis B-associated HCC (7). Additionally, Forns index predicts recurrence and death in patients with HCC after a curative resection (8), and some evidence has shown that the S-index (a simpler mathematical model) is related to the degree of liver cirrhosis and fibrosis (9). However, there are few studies comparing the prognostic value of liver function markers and exploring a reliable model (including complete liver function markers and pathological characteristics) for HCC patients. Nomograms have shown excellent performance in the prediction of colon cancer and other tumors (10, 11). In this study, a nomogram was built to predict the overall survival of HCC patients.

## Materials and Methods

### Patient Inclusion and Exclusion Criteria

Blood tests and clinical data were collected from 560 HCC patients who were hospitalized at the First Affiliated Hospital of Anhui Medical University between January 2007 and January 2014. These patient data were used for the training and validation of the proposed model. Basic information was also received from 180 HCC patients who were hospitalized at the First Affiliated Hospital of Wenzhou Medical University between January 2014 and January 2016, and these patient data were used for additional external validation of the proposed model. This research was approved by the Ethical Committee and Institutional Review Board of the First Affiliated Hospital of Anhui Medical University and the First Affiliated Hospital of Wenzhou Medical University. Signed informed consent was obtained from every patient.

Patient data were analyzed retrospectively for this study. The inclusion criteria included: 1) confirmed HCC diagnosis by pathology; 2) definite surgery; 3) peripheral blood tests obtained within one week prior to surgery; and 4) signed informed consent. The exclusion criteria included; 1) previous malignancies or various primary tumors; 2) undergone radiation treatment or chemotherapy previously prior to the surgery; 3) the presence of certain diseases that could influence peripheral blood cell counts; 4) HCC patients that could not undergo curative resection, such as those with distant metastases; and 5) patients with severe diseases that prevent them from having surgery, such as severe vital organ damage. A final cohort of 560 from the original 812 patients with HCC were included in this study for analysis. All cases were randomly grouped into either the training set or validation set at a 7:3 ratio using a computerized method.

### Data Collection and Follow-Up

Patient demographic and clinical pathology data were gathered at the medical record room of our hospital. The information that was collected included: age, gender, body mass index (BMI), tumor size, number of tumors, differentiation grade, presence or absence of cirrhosis, history of alcohol abuse, portal hypertension, microvascular invasion, invasion of adjacent tissue, portal vein tumor thrombus (PVTT), intrahepatic transfer and test results of the surface antigen of the hepatitis B virus (HBsAg). PVTT (which indicates that the liver tumor has invaded the portal vein system) was diagnosed by computed tomography (CT), magnetic resonance imaging (MRI) or ultrasound. Routine laboratory data that were collected included: albumin, alpha fetoprotein (AFP), total bilirubin, total cholesterol, prothrombin time, alanine aminotransferase (ALT), aspartate aminotransferase (AST), gamma-glutamyl transpeptidase (GGT), fibrinogen and platelet counts. Before surgery, the disease progression of each HCC patient was evaluated according to CT and other techniques. Based on these indexes, the stage of HCC was determined according to the classification systems of Child–Pugh, BCLC, TNM, Okuda and CLIP.

Among the 560 HCC patients, the number of right hepatocellular tumors and left hepatocellular tumors are 386 and 174, respectively; 254 tumors are larger than 5cm, the median tumor size is 4cm; Among the patients, 477 cases underwent a major liver resection, and 83 cases had a minor liver resection. The number of open surgeries and laparoscopic surgeries were 345 and 215, respectively.

The patients who were enrolled received prospective follow-up. The follow-up visits were obtained either by telephone or outpatient visits, and were carried out at regular intervals (every 90 days within the first 2 years following surgery, every 180 days between 3 and 5 years post-surgery, and once a year after 5 years). Three-year follow-up information was also obtained from the external group of patients at the other medical center.

### Statistical Analysis

Continuous variables were expressed as mean ± standard deviation (SD) and data were analyzed using the Student’s *t*-test. Categorical variables were expressed as number (percent) and compared using either a Chi-square test or Fisher’s exact test. The univariate and multivariate analyses were carried out using the Cox proportional hazards model. The Kaplan–Meier survival curves were compared using the log-rank test. Harrell’s C-index (concordance index) was used in the nomogram to evaluate the performance of the model. Time-dependent receiver operating characteristic (tdROC) curves and calibration curves were used to verify the accuracy of the novel scoring system in the nomogram. All data analyses was carried out using SPSS (16.0 version) and R Studio software (version 1.1.447).

## Results

### Baseline Characteristics

Analysis of the baseline characteristics from 560 patients (392 from the training dataset and 168 from the validation dataset) revealed that most of the variables were not statistically different (p > 0.05) between the training and validation groups (**Table 1**). The baseline demographics and clinical characteristics of patients in external cohort are showed in the **Supplementary Table 1**.

**Table 1:** Baseline demographics and clinical characteristics of patients in training cohort and validation cohort.

Variable	Training set (n = 392)	Validation set (n = 168)	p value
Gender			0.093
Female	68 (17.3)	19 (11.3)	
Male	324 (82.7)	149 (88.7)	
Age (years)	55.90 ± 11.31	56.10 ± 11.57	0.851
BMI (kg/m^2^)	22.73 ± 3.29	22.85 ± 2.63	0.681
Cirrhosis			0.969
Yes	266 (67.9)	113 (67.3)	
No	126 (32.1)	59 (32.7)	
Tumor size (cm)	5.32 ± 3.62	4.77 ± 3.73	0.105
Portal vein tumor thrombus			1.000
Yes	15 (3.8)	7 (4.2)	
No	377 (96.2)	161 (95.8)	
Invasion of adjacent tissues			0.190
Yes	16 (4.1)	12 (7.1)	
No	376 (95.9)	156 (92.9)	
Microvascular invasion			0.228
Yes	56 (14.3)	17 (10.1)	
No	336 (85.7)	151 (89.9)	
Intrahepatic transfer			1.000
Yes	85 (21.7)	36 (21.4)	
No	307 (78.3)	132 (78.6)	
Tumor grade			0.335
Low	279 (71.2)	112 (66.7)	
High	113 (28.8)	56 (33.3)	
HBsAg			0.689
Yes	320 (81.6)	134 (79.8)	
No	72 (18.4)	34 (20.2)	
Alpha fetoprotein (ng/mL)	2120.89 ± 8730.20	1901.61 ± 7733.66	0.778
Prothrombin time (s)	14.03 ± 1.16	14.30 ± 1.42	0.017
Fibrinogen (g/L)	3.09 ± 1.01	3.10 ± 1.52	0.942
Platelet (10*^9^/L)	150.41 ± 65.93	133.08 (67.69)	0.005
Albumin (g/L)	39.31 ± 5.73	39.25 ± 5.65	0.908
Total bilirubin (μmol/L)	13.71 ± 8.70	14.05 ± 8.75	0.673
Total cholesterol (mmol/L)	4.29 ± 1.09	4.23 ± 1.03	0.586
Alanine aminotransferase (U/L)	53.08 ± 61.80	43.82 ± 40.60	0.075
Aspartate aminotransferase (U/L)	97.16 ± 166.75	79.54 ± 101.44	0.204
γ-glutamyl transpeptidase (U/L)	96.80 ± 150.49	88.83 ± 95.62	0.527
TNM stage			0.500
I–II	55 (14.0)	28 (16.7)	
III–IV	337 (86.0)	140 (83.3)	
Child-Pugh			0.772
A	270 (68.9)	118 (70.2)	
B	122 (31.1)	50 (29.8)	
CLIP			0.395
0	116 (29.6)	62 (36.9)	
1	95 (24.2)	41 (24.4)	
2	91 (23.2)	35 (20.8)	
3	57 (14.5)	17 (10.1)	
4-5	33 (8.4)	13 (7.7)	
Okuda			0.082
I	197 (50.3)	100 (59.5)	
II	183 (46.7)	66 (39.3)	
III	12 (3.1)	2 (1.2)	
BCLC			0.623
A	279 (71.2)	126 (75.0)	
B	50 (12.8)	16 (9.5)	
C	55 (14.0)	24 (14.3)	
D	8 (2.0)	2 (1.2)	

Continuous variables were expressed by mean ± standard deviation. Abbreviations: BCLC, the Barcelona Clinic Liver Cancer staging system; Okuda, the Okuda staging system; CLIP, the Cancer of the Liver Italian Program; TNM stage, the seventh edition of the TNM system; HBsAg, surface antigen of the hepatitis B virus; BMI, body mass index.

### Prognostic Factors of the Training Cohort

Univariate risk factors of overall survival (OS) are shown in **Table 2**. The results showed that classifications of Child–Pugh, BCLC, TNM, Okuda and CLIP, as well as tumor size, PVTT, invasion of adjacent tissues, microvascular invasion, intrahepatic transfer, tumor grade, prothrombin time, and levels of fibrinogen, albumin, total bilirubin, total cholesterol, ALT and GGT were significant indicators of OS. These significant indicators from the univariate analysis (p values less than 0.05), but not the other classification systems, were included in the multivariate analysis to build a simple nomogram. The results showed that tumor size, PVTT, invasion of adjacent tissues, microvascular invasion, fibrinogen and total bilirubin were independent prognostic factors for OS (**Table 3**).

**Table 2:** Univariate analysis of the training cohort.

	beta	HR (95% CI for HR)	Wald test	p value
Gender	-0.06545	0.9366 (0.645–1.36)	0.12	0.731
Age	-0.002542	0.9975 (0.9856–1.01)	0.17	0.6784
Body mass index	-0.0198	0.9804 (0.9364–1.026)	0.72	0.3975
History of alcohol abuse	0.084	0.920 (0.720–1.175)	0.450	0.503
Cirrhosis	0.07483	1.078 (0.8001–1.452)	0.24	0.6224
Portal hypertension	0.23	1.253 (0.9201–1.706)	2.05	0.152
Tumor size	0.1337	1.143 (1.105–1.183)	59.18	1.437e-14
Portal vein tumor thrombus	1.764	5.835 (3.337–10.2)	38.27	6.153e-10
Invasion of adjacent tissues	1.18	3.254 (1.884–5.621)	17.91	2.312e-05
Microvascular invasion	0.7721	2.164 (1.529–3.063)	18.98	1.318e-05
Intrahepatic transfer	0.6371	1.891 (1.391–2.571)	16.53	4.782e-05
Tumor grade	0.3624	1.437 (1.073–1.924)	5.91	0.01507
HBsAg	0.3291	1.39 (0.9425–2.049)	2.76	0.0967
Alpha fetoprotein	9.514e-06	1 (1–1)	2.32	0.1275
Prothrombin time	0.1943	1.214 (1.089–1.354)	12.28	0.0004571
Fibrinogen	0.26	1.297 (1.136–1.481)	14.79	0.0001199
Platelet	-0.0016	0.9984 (0.9961–1.001)	1.88	0.1705
Albumin	-0.06593	0.9362 (0.9158–0.957)	34.47	4.328e-09
Total bilirubin	0.03502	1.036 (1.022–1.049)	27.22	1.82e-07
Total cholesterol	-0.1711	0.8428 (0.732–0.9703)	5.66	0.01734
Alanine aminotransferase	0.002376	1.002 (1.001–1.004)	9.4	0.002166
Child Pugh	0.7416	2.099 (1.625–2.712)	32.19	1.399e-08
CLIP	0.3715	1.45 (1.304–1.612)	47.22	6.335e-12
Okuda	0.8141	2.257 (1.742–2.925)	37.86	7.602e-10
BCLC	0.4685	1.598 (1.369–1.864)	35.34	2.764e-09
Aspartate aminotransferase	0.0002021	1 (0.9995–1.001)	0.3	0.5838
γ-glutamyl transpeptidase	0.0005726	1.001 (1–1.001)	4.73	0.02968
TNM stage	1.252	3.499 (2.502–4.892)	53.59	2.466e-13

HR, hazard ratio; CI, confidence interval; BCLC, the Barcelona Clinic Liver Cancer staging system; Okuda, the Okuda staging system; CLIP, the Cancer of the Liver Italian Program; TNM stage, the seventh edition of the TNM system; HBsAg, surface antigen of the hepatitis B virus.

**Table 3:** Multivariate analysis of the training cohort.

	coef	exp(coef)	se(coef)	z	p
Tumor size	0.071	1.073	0.022	3.278	0.001
Portal vein tumor thrombus	1.219	3.384	0.310	3.936	0.000
Invasion of adjacent tissues	0.696	2.005	0.309	2.251	0.024
Microvascular invasion	0.537	1.711	0.230	2.335	0.020
Intrahepatic transfer	0.133	1.142	0.204	0.654	0.513
Prothrombin time	0.116	1.123	0.082	1.411	0.158
Fibrinogen	0.162	1.176	0.080	2.039	0.041
Albumin	-0.024	0.976	0.015	-1.543	0.123
Total bilirubin	0.019	1.020	0.008	2.462	0.014
Total cholesterol	-0.058	0.944	0.073	-0.801	0.423
Alanine aminotransferase	0.002	1.002	0.001	1.537	0.124
γ -glutamyl transpeptidase	0.000	1.000	0.000	0.171	0.865

### Prognostic Nomogram for Survival

**Figure 1:**
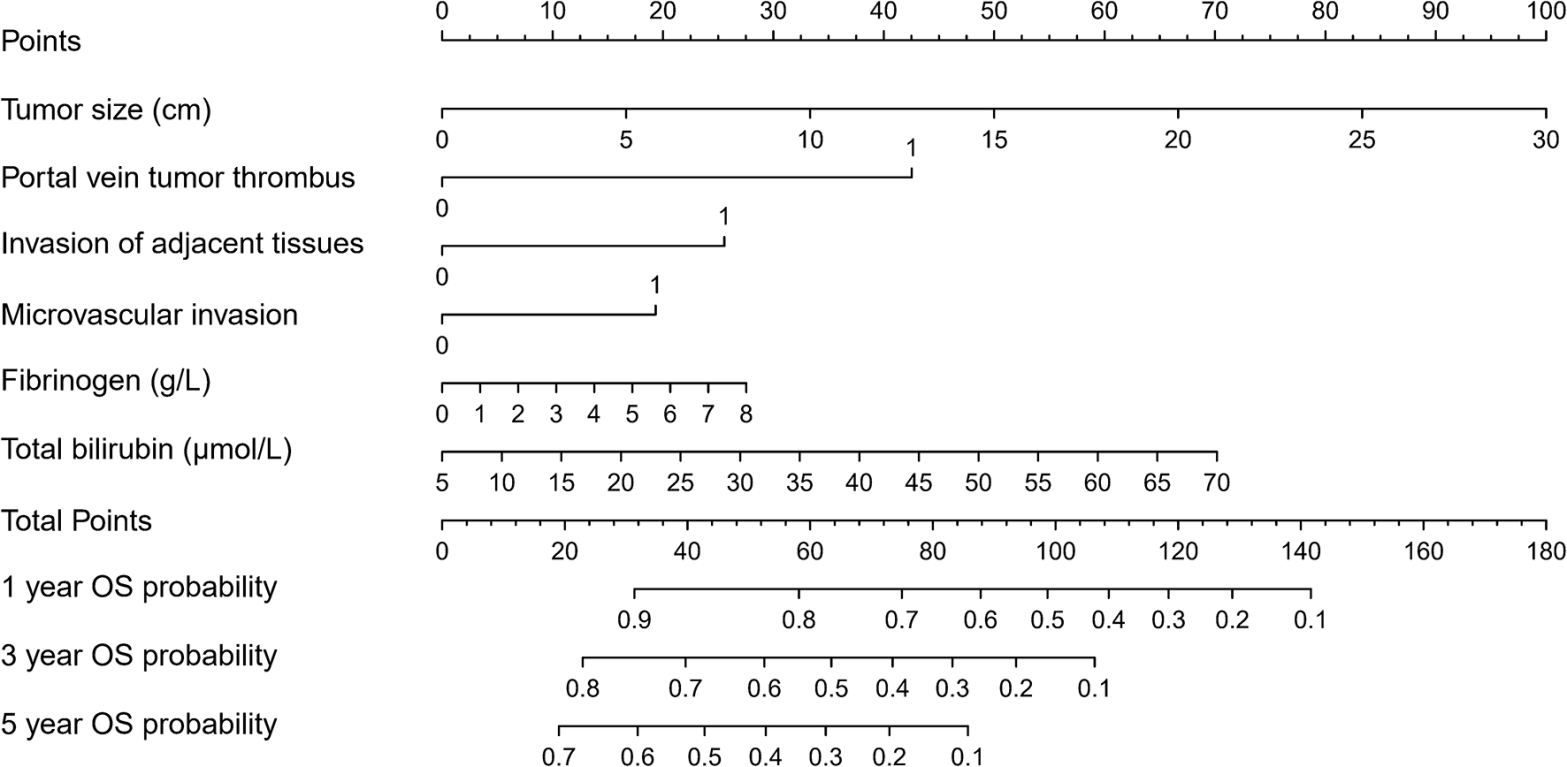
Nomogram for predicting overall survival (OS) after curative resection of livers in patients with hepatocellular carcinoma (HCC).

Based on the Cox regression model, a nomogram was constructed to predict OS of patients with HCC (**Figure 1**). To construct this nomogram, each subgroup variable was assigned a corresponding score according to the specific situation of each HCC patient. A point system was used to assign a score ranging from 0 to 100 to each subgroup variable. Then, the corresponding 1-, 3-, and 5-year OS rates were predicted. The nomogram scoring system is presented in **Table 4**.

**Table 4:** Nomogram Scoring System.

Tumor size	points	PVTT	points	Fibrinogen	points	Iat	Points	MiP	Points	Tb	Points
0	0	0	0	0	0	0	0	0	0	5	0
5	17	1	43	1	3	1	26	1	19	10	5
10	33			2	7					15	11
15	50			3	10					20	16
20	67			4	14					25	22
25	83			5	17					30	27
30	100			6	21					35	32
				7	24					40	38
				8	28					45	43
										50	49
										55	54
										60	59
										65	65
										70	70
**Total points**	**1-Year Survival probability**		**Total points**		**3-Year Survival probability**		**Total points**		**5-Year Survival probability**		
142	0.1		106		0.1		86		0.1		
142	0.1		106		0.1		86		0.1		
129	0.2		94		0.2		73		0.2		
118	0.3		83		0.3		63		0.3		
109	0.4		73		0.4		53		0.4		
99	0.5		63		0.5		43		0.5		
88	0.6		53		0.6		32		0.6		
75	0.7		40		0.7		19		0.7		
58	0.8		23		0.8						
31	0.9										

PVTT, Portal vein tumor thrombus (0: no , 1: yes); Iat, Invasion of adjacent tissues (0: no, 1: yes); MiP, Microvascular invasion (0: no, 1: yes); Tb, Total bilirubin.

### Validation of the Nomogram

**Figure 2:**
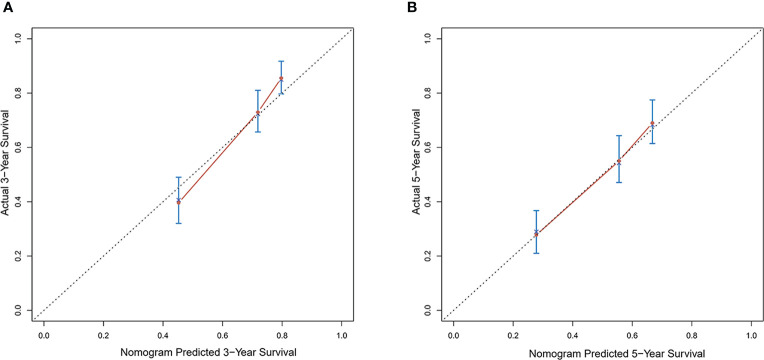
Calibration curves of the prognostic monogram for the **(A)** 3-year overall survival (OS) and **(B)** 5-year OS in the training dataset.

**Figure 3:**
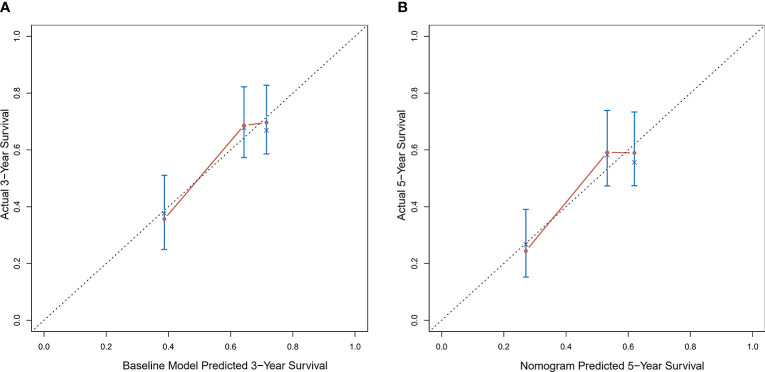
Calibration curves of the prognostic nomogram for the **(A)** 3-year overall survival (OS) and **(B)** 5-year OS in the validation dataset.

**Figure 4:**
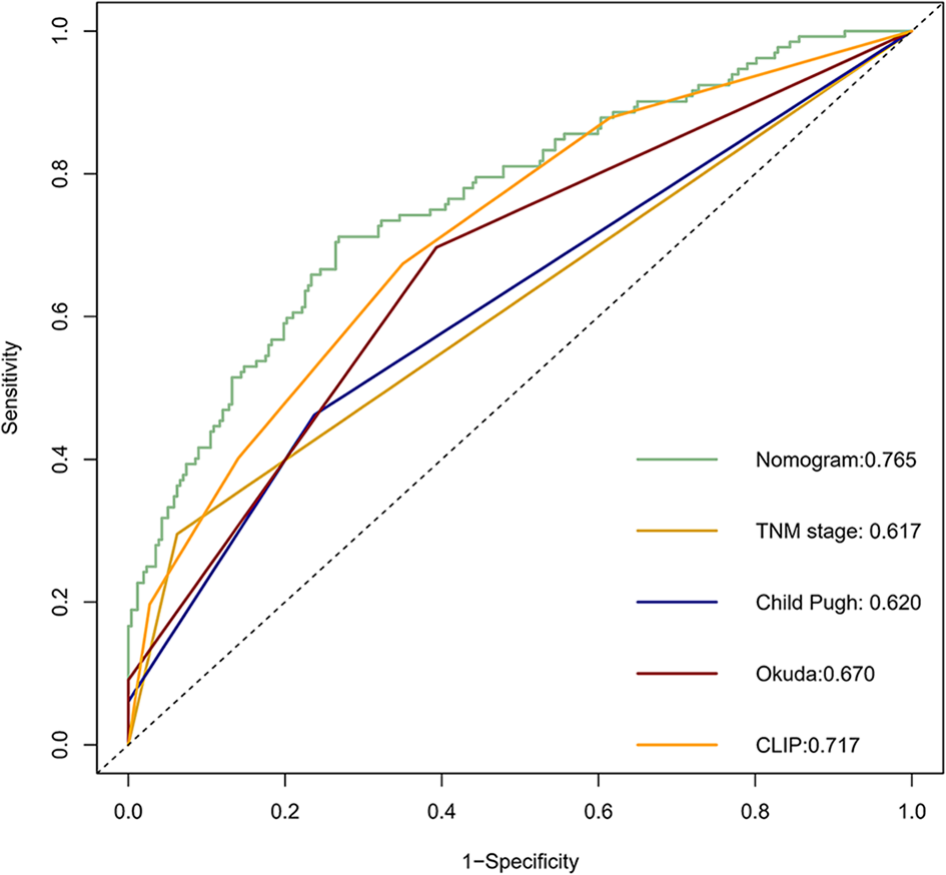
The receiver operating characteristic (ROC) curve of the prognostic nomogram in the training dataset compared with other systems.

**Figure 5:**
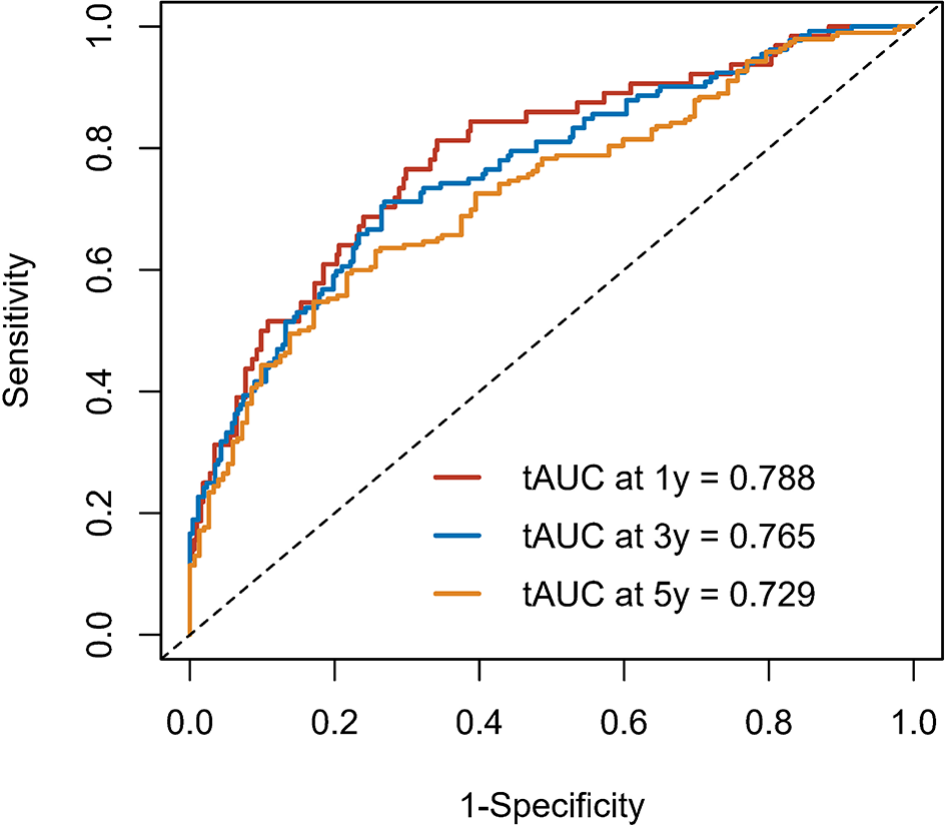
The time-dependent receiver operating characteristic (tdROC) curve of the prognostic nomogram in the training group.

**Figure 6:**
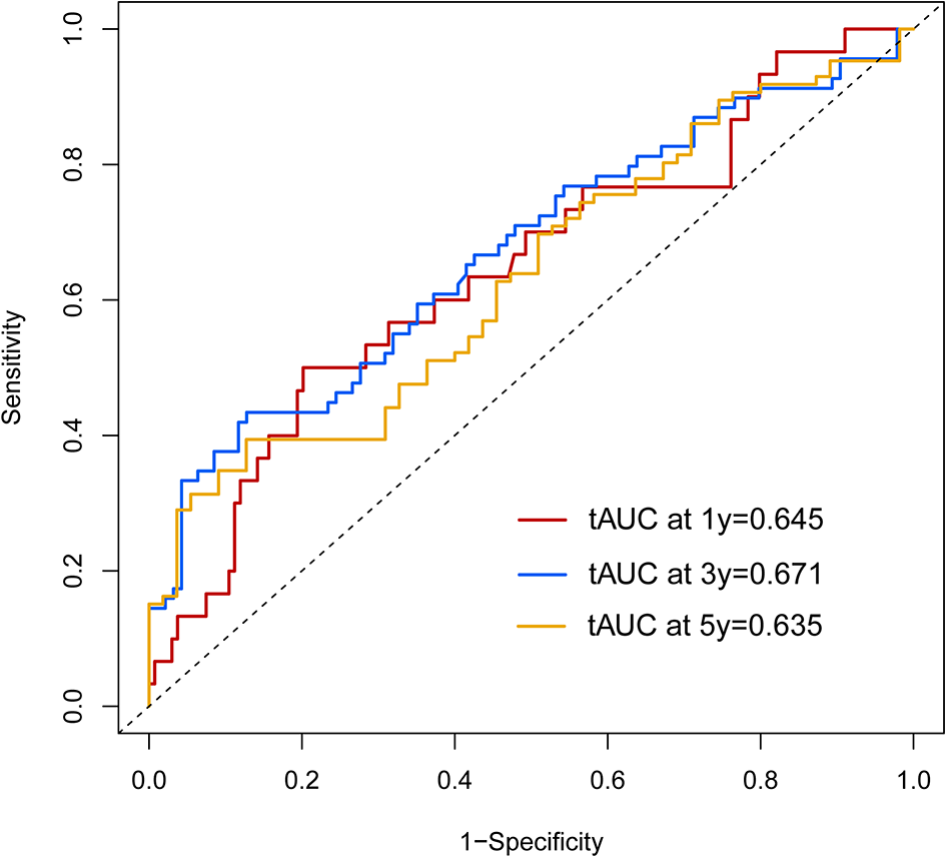
The time-dependent receiver operating characteristic (tdROC) curve of the prognostic nomogram in the validation group.

Calibration curves were used to verify the performance of the model in predicting OS of HCC patients (**Figures 2** and **3**). The C-index of the model in the training group was 0.695 (95% confidence interval [CI]: 0.644–0.746), and in the validation group was 0.672 (95% CI: 0.592–0.753). To further validate the performance of the model, the ROC curve was plotted for the nomogram and compared to the other scoring systems (**Figure 4**). The area under the ROC curve (AUC-ROC) of the nomogram was larger than those from the Child-Pugh classification, BCLC, TNM, Okuda and CLIP. Additionally, the tdROC curves of the nomogram (1, 3, and 5 years) were plotted for the training and validation groups (**Figures 5** and **6**), and the AUCs indicated good performance of the predictive model.

### Kaplan-Meier Survival Curves

**Figure 7:**
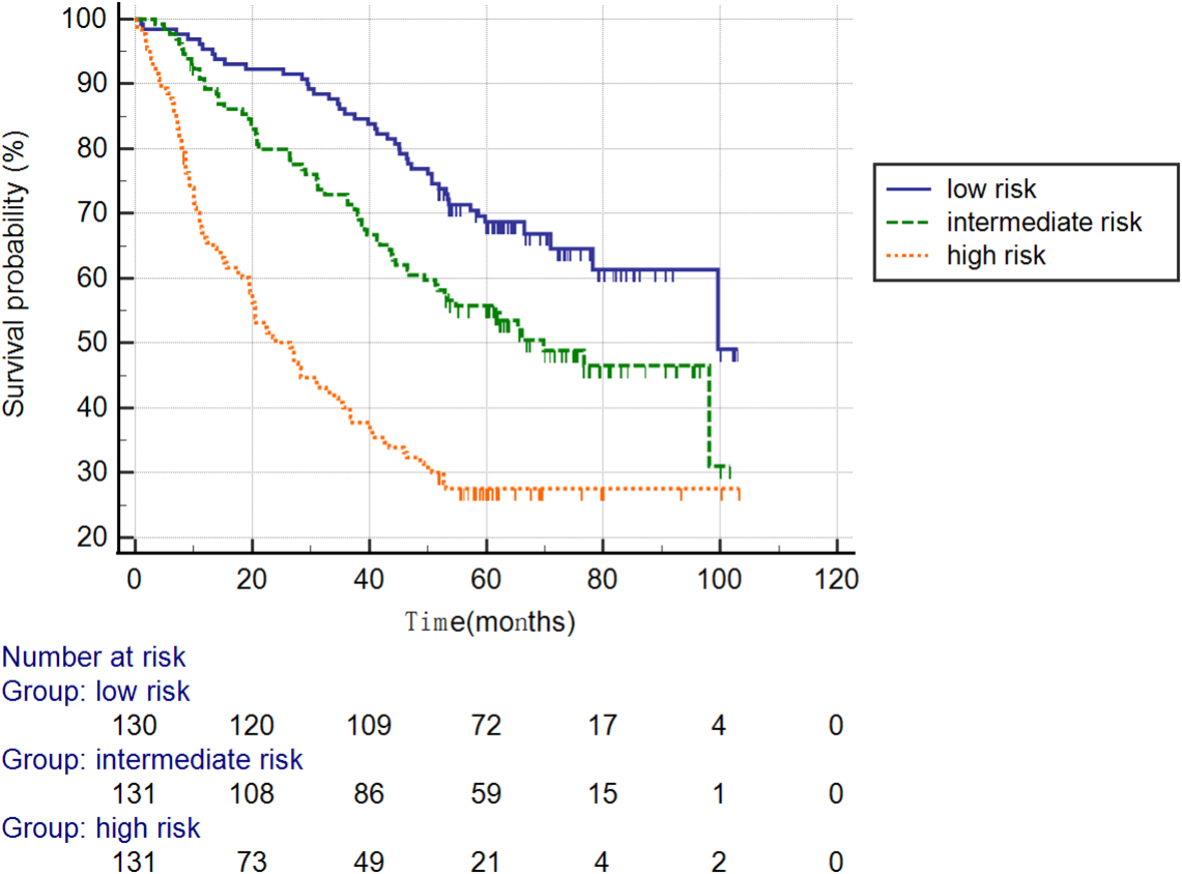
Survival curves stratified by the score calculated using the nomogram in the training cohort (low risk: < 30; intermediate risk: 30–50; and high risk: > 50).

Furthermore, the patients were stratified into three subgroups based on cutoff-values of the nomogram (low risk: < 30; intermediate risk: 30–50; and high risk: > 50; **Figure 7**). The Kaplan–Meier curves revealed excellent prediction results of this novel scoring model.

### External Validation of the Developed Model

**Figure 8:**
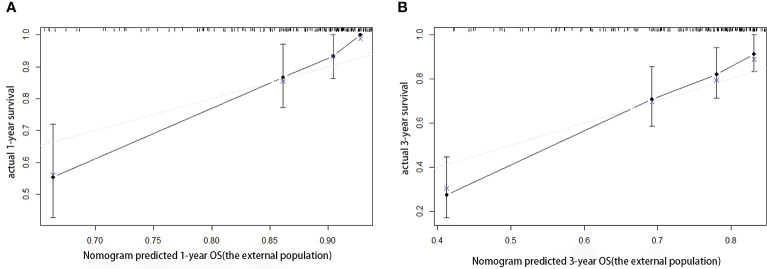
Calibration curves of the prognostic nomogram for the **(A)** 1-year overall survival (OS) and **(B)** 3-year OS in the external population.

**Figure 9:**
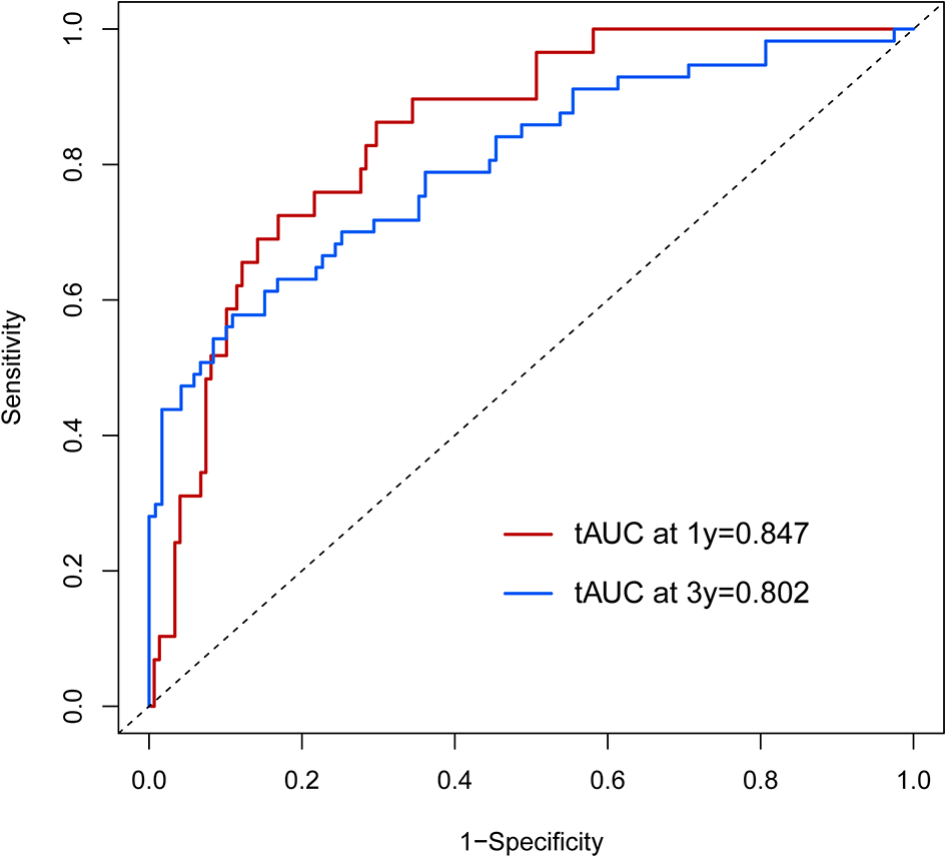
The time-dependent receiver operating characteristic (tdROC) curve of the prognostic nomogram in the external population.

According to the scoring of the nomogram developed with the training group, the 1-year (**Figure 8**) and 3-year (**Figure 8**) calibration curves as well as the tdROC curve (**Figure 9**) of the external test population were analyzed. The C-index was 0.718 (95% CI: 0.565–0.944). These results indicate that the results from the external group were highly consistency with those of the training group.

## Discussion

Surgery is considered the only means of curative treatment for HCC. Due to the limitation of diagnostic techniques, it is often difficult to detect HCC early, which leads to a poor prognosis in many patients (3). Therefore, in an effort to improve the prognosis of HCC, many scholars have made considerable contributions to the field. There separate prognostic systems, TNM stage, BCLC and CLIP, were defined but these models are complex and difficult to use. Therefore, studies to develop a simple prognostic scoring system have been widely performed in recent years. Certain studies have shown that elevated levels of serum markers especially liver function indexes, may be associated with the poorer prognosis of HCC patients. For example, ALT, AST, and GGT are significantly related to the survival of HCC patients (12). To the best of our knowledge, this study is the first attempt to develop a prognostic nomogram that combined regular serum markers (complete liver function indexes) and clinical pathology characteristics to estimate the probability of 1-, 3,- and 5-year OS rates. This novel prognostic nomogram was more accurate in making predictions for the prognosis of HCC compared with the other prognostic systems.

Based on the results of the multivariate analysis, tumor size, PVTT, invasion of adjacent tissues, microvascular invasion, fibrinogen and total bilirubin were independent prognostic factors for OS. Therefore, we developed a nomogram using these markers. The C-index was 0.695, which indicated that this new model is accurate in predicting the prognosis of HCC. Moreover, the C-index from the analysis of the external group was 0.718, and the tdROC curve and calibration curves revealed excellent outcomes in the training and validation groups. Therefore, this nomogram based on multiple factors including serum components and pathology characteristics has reliable prognostic value for HCC patients.

In recent years, nomograms have shown high reliability for predicting tumor prognosis as a statistical model. Importantly, nomograms have better value in predicting prognosis than TNM stage in many cancers (13, 14). This model has been identified as a new standard, and our study reached the same conclusion: the AUC of the nomogram was larger than the AUCs for the Child–Pugh, TNM stage, BCLC and CLIP classification systems. This nomogram is composed of simple markers that can be easily applied in a clinic setting. A point was used to assign a value between 0 and 100 for each variable for each HCC patient after surgery. This scoring system involves calculating the total points and predicting the 1- and 3-year survival rates according to the nomogram, so the novel nomogram is useful to assess the survival of patients that have undergone liver resection and help decide on the appropriate treatment. For example, high risk scores of patients should have more attention given to follow-up and adequate treatment strategies after the surgery.

Six independent risk factors were included in the nomogram. Tumor size, invasion of adjacent tissues and microvascular invasion were pathological characteristics that can reflect the progression of the disease, and these results were consistent with previous studies (15–17). Additionally, PVTT, fibrinogen and total bilirubin were also independent risk factors. PVTT is a serious problem that can affect the prognosis of cancer patients (18), and patients with PVTT have high recurrence rates (19). A high level of fibrinogen in the serum can lead to the occurrence of PVTT, and a meta-analysis has verified that elevated fibrinogen levels are associated with poor prognosis and advanced tumor progression (20). Total bilirubin, another serum biomarker, has been shown in some studies to play a role in predicting the survival of HCC patients (21, 22). Taken together, these indications should be included as standard markers of disease in HCC patients.

Our research has certain limitations that need to be considered. Firstly, the external population requires additional cases from many other centers in other countries to verify the results, and secondly, the C-index needs to be improved with these additional center studies.

In summary, tumor size, invasion of adjacent tissues, microvascular invasion, PVTT, fibrinogen, and total bilirubin levels were risk factors for the prognosis of HCC patients, and the novel nomogram model developed from these factors has reliable prognostic value for HCC patients.

## Data Availability Statement

The data used to support the findings of this study are available from the corresponding author upon request.

## Ethics Statement

The studies involving human participants were reviewed and approved by Institutional Review Board and the Ethics Committee of the First Affiliated Hospital of Anhui Medical University and the First Affiliated Hospital of Wenzhou Medical University. Written informed consent was obtained from the individual(s) for the publication of any potentially identifiable images or data included in this article.

## Author Contributions

L-xZ revise the main work. A-mX, PX and JX designed this study, P-qL, L-C and D-dS help revised this manuscript. All authors contributed to the article and approved the submitted version.

## Conflict of Interest

The authors declare that the research was conducted in the absence of any commercial or financial relationships that could be construed as a potential conflict of interest.

## Abbreviations

HCC, hepatocellular carcinoma; C-index, concordance index; OS, overall survival; PVTT, portal vein tumor thrombus; ROC, receiver operating characteristic; tdROC, time-dependent receiver operating characteristic; AUC, area under the curve; ALT, alanine aminotransferase; AST, aspartate aminotransferase; GGT, gamma-glutamyl transpeptidase; BCLC, Barcelona Clinic Liver Cancer staging system; Okuda, Okuda staging system; CLIP, the Cancer of the Liver Italian Program; TNM, seventh edition of the tumor node metastasis) system.
