# Supplementary material for: Model to Predict Overall Survival in Patients With Hepatocellular Carcinoma After Curative Hepatectomy
Source: Front Oncol. 2021 Mar 5;10:537526. doi: 10.3389/fonc.2020.537526 (PMC7977285; doi:10.3389/fonc.2020.537526)
Supplement: Supplementary file 1 [file Table_1.doc]

**Supplementary Table 1. Baseline demographics and clinical characteristics of patients in external cohort**

| **Variable** | **External set (n = 180)** |
| --- | --- |
| Gender |  |
| Female | 33 (18.3) |
| Male | 147 (81.7) |
| Age (years) | 57.62 ± 10.93 |
| BMI (kg/m^2^) | 22.64 ± 3.37 |
| Cirrhosis |  |
| Yes | 136 (75.6) |
| No | 44 (24.4) |
| Tumor size (cm) | 5.67 ± 3.88 |
| Portal vein tumor thrombus |  |
| Yes | 10 (5.6) |
| No | 170 (94.4) |
| Invasion of adjacent tissues |  |
| Yes | 9 (5.0) |
| No | 171 (95.0) |
| Microvascular invasion |  |
| Yes | 30 (16.7) |
| No | 150 (83.3) |
| Intrahepatic transfer |  |
| Yes | 52(28.9) |
| No | 128 (71.1) |
| Tumor grade |  |
| Low | 123 (68.3) |
| High | 57 (31.7) |
| HBsAg |  |
| Yes | 140 (77.8) |
| No | 40 (22.2) |
| Alpha fetoprotein (ng/mL) | 1822.33 ± 7373.57 |
| Prothrombin time (s) | 14.13 ± 1.27 |
| Fibrinogen (g/L) | 3.08 ± 1.09 |
| Platelet (10*9/L) | 134.41 ± 66.34 |
| Albumin (g/L) | 38.91 ± 5.73 |
| Total bilirubin (μmol/L) | 13.28 ± 7.89 |
| Total cholesterol (mmol/L) | 4.29 ± 1.17 |
| Alanine aminotransferase (U/L) | 48.61 ± 45.82 |
| Aspartate aminotransferase (U/L) | 58.66 ± 68.20 |
| γ-glutamyl transpeptidase (U/L) | 130.49 ± 207.68 |
| TNM stage |  |
| I - II | 36 (20.0) |
| III - IV | 144 (80.0) |
| Child-Pugh |  |
| A | 116 (64.4) |
| B | 64 (35.6) |
| CLIP |  |
| 0 | 51 (28.3) |
| 1 | 39 (21.7) |
| 2 | 41 (22.8) |
| 3 | 30 (16.7) |
| 4-5 | 19 (10.5) |
| Okuda |  |
| I | 72 (40.0) |
| II | 101 (56.1) |
| III | 7 (3.9) |
| BCLC |  |
| A | 126 (70.0) |
| B | 22 (12.2) |
| C | 26 (14.4) |
| D | 6 (3.3) |

Continuous variables were expressed by mean ± standard deviation. Abbreviations: BCLC, the Barcelona Clinic Liver Cancer staging system; Okuda, the Okuda staging system; CLIP, the Cancer of the Liver Italian Program; TNM stage, the seventh edition of the TNM system; HBsAg, surface antigen of the hepatitis B virus; BMI, body mass index.
